# Identification of *Drosophila melanogaster* as a model organism in COPD research

**DOI:** 10.3389/fimmu.2026.1707110

**Published:** 2026-02-12

**Authors:** Yuan Zhang, Jing Kou, Yuxuan Zhang, Haiyi Huang, Yang Yang, Chengshuang Liu, Ting Lu, Xiaoxuan Zhao, Yongqi Liu, Jianzheng He

**Affiliations:** 1 College of Basic Medicine, Gansu University of Chinese Medicine, Lanzhou, China; 2 Provincial-level Key Laboratory for Molecular Medicine of Major Diseases and The Prevention and Treatment with Traditional Chinese Medicine Research in Gansu Colleges and University, Gansu University of Chinese Medicine, Lanzhou, China; 3 Key Laboratory for Transfer of Dunhuang Medicine at the Provincial and Ministerial Level, Gansu University of Chinese Medicine, Lanzhou, China; 4 College of Integrative Medicine, Gansu University of Chinese Medicine, Lanzhou, China; 5 College of Veterinary Medicine, Gansu Agricultural University, Lanzhou, China

**Keywords:** COPD models, COPD pathogenesis, *Drosophila melanogaster*, genetic characteristics, therapies

## Abstract

Chronic obstructive pulmonary disease (COPD) is a progressive respiratory condition characterized by increasing mortality and morbidity. Current animal models have certain limitations in elucidating the pathophysiology and underlying mechanisms of COPD, which hinder effective treatments. There is an urgent need to identify an informative model that can dissect the COPD mechanisms and screen therapeutic drugs. The *Drosophila melanogaster* is regarded as an ideal *in vivo* model for studying COPD due to its ability to present representative pathological hallmarks within a short time frame, its visualized tracheal morphology, and well-established genetic tools. In this study, we explore the feasibility of using *Drosophila* as a novel invertebrate model for investigating COPD. We summarize the conserved features between flies and mammals in response to airway inflammation, including airway structures, pathophysiological changes, immune responses, molecular mechanisms, and modeling approaches. Additionally, we outline potential translational applications, including high-throughput identification, drug discovery, and a prioritized preclinical platform. We also propose integrating insights from *Drosophila* with mammalian models and clinical COPD endotypes.

## 1 Introduction

Chronic obstructive pulmonary disease (COPD) is a chronic respiratory condition (1). Approximately 4 million individuals worldwide die from COPD each year, with causes including cigarette smoke, air pollutants, occupational hazards, biomass exposure, genetic predispositions, and aging-related inflammatory responses. Alarmingly, its incidence is projected to rise (2). While hereditary factors, viruses, and bacteria play significant roles in driving COPD, the primary trigger is believed to be the inhalation of cigarette smoke (3, 4). Histopathologically, COPD is characterized by chronic bronchitis, persistent airflow limitation, and irreversible respiratory obstruction, even after smoking cessation. Pulmonary tissue experiences inflammation, oxidative stress, and dysfunction of the protease–antiprotease balance, leading to COPD (5, 6). COPD has been characterized by distinct clinical phenotypes and endotypes (7). Its typical phenotype of COPD progression is marked by chronic inflammation, accompanied by pro-inflammatory substances and inflammatory cells (8, 9). Until now, COPD remains difficult to treat. Glucocorticoid therapy is one of the chief treatment options for COPD; however, it is often associated with drug tolerance and adverse reactions, limiting its effectiveness (2). To further understand the molecular mechanisms underlying COPD progression and to develop effective intervention and prevention strategies, there is an urgent need for a straightforward, user-friendly, and efficient animal model.

**Figure 1:**
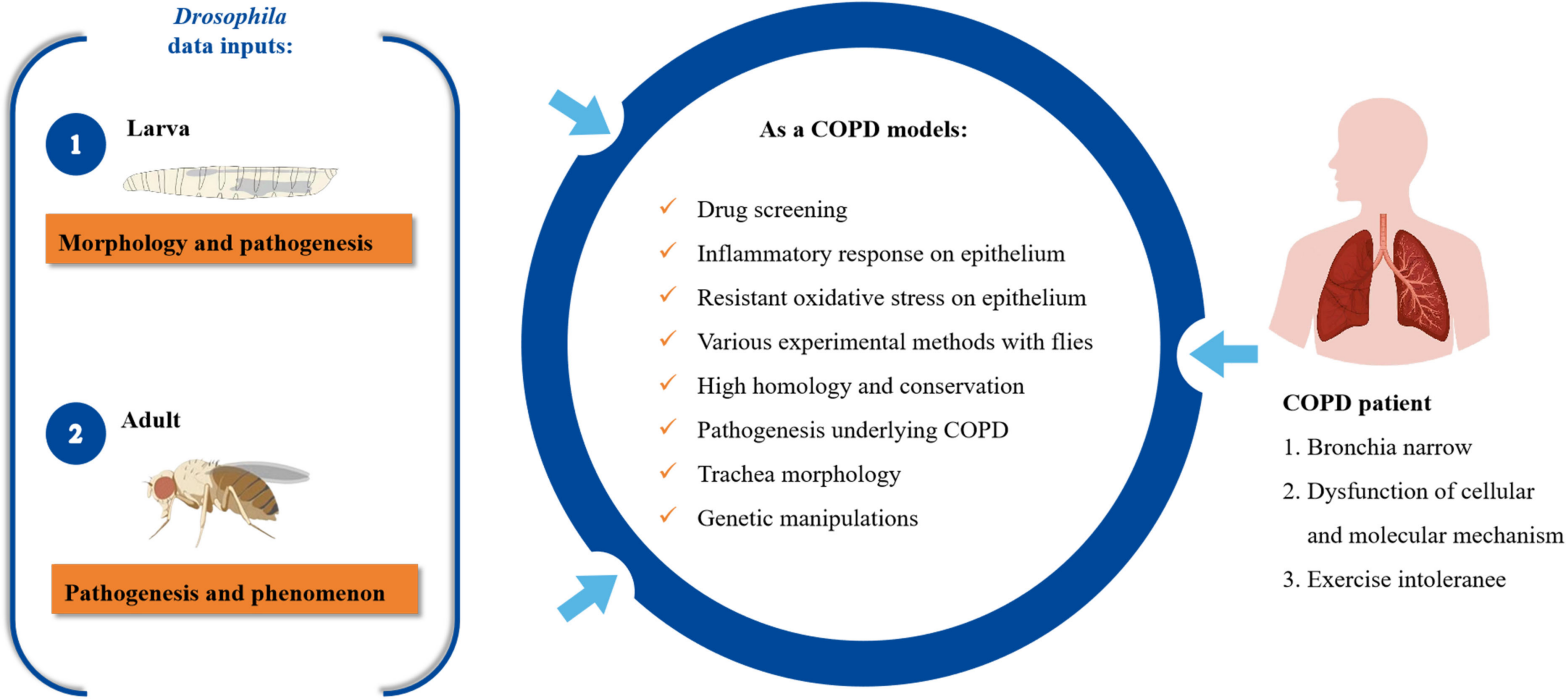
*Drosophila melanogaster* as a COPD model organism. Based on genes, homologs, molecular mechanisms, and organizational structure in fly airway; capable of serving as a favorable model for studying chronic inflammation, oxidative stress, drug screening, and susceptibility factors that underlie COPD. COPD, chronic obstructive pulmonary disease.

Animal models are widely recognized as invaluable tools for advancing our understanding of the onset and treatment of human diseases. Utilizing representative models is particularly beneficial for accurately replicating conditions associated with COPD, such as emphysema, airway fibrosis, and remodeling. Mammalian models—including rodents, beagles, miniature pigs, and rhesus monkeys—have been employed to study COPD. However, these models have certain limitations (**Table 1**) (10–12); they involve high maintenance costs, raise ethical concerns, and also fail to carry out high-throughput screening of pathogenic genes and pharmacological efficacy. The *Drosophila melanogaster*, commonly known as the fruit fly, has been extensively used as a powerful tool for screening candidate treatments for various human diseases, including neurodegenerative disorders, cardiovascular diseases, inflammation, infectious diseases, cancer, and metabolic disorders (13–15). Flies share notable similarities with the human organs and systems, such as the brain, heart, bronchi, and gut. Approximately 75% of disease-related genes and 80% of conserved functional protein domains are conserved homologs between humans and flies (13, 16). The clear genetic background and simple physiological structure of the fly allow its organs, tissues, and cells to be easily visualized and labeled. These characteristics provide new insights into pathogenesis, disease genes, and treatments—areas where mammalian models offer less interpretability (**Figure 1**). Given these advantageous traits, combined with advanced biological techniques, *Drosophila* has become a valuable model for conducting analytical rather than merely descriptive studies on human diseases.

**Table 1:** Advantages and disadvantages of COPD animal models.

Models	Advantage	Disadvantage	References
Mice	• Strong operability • Small size • Conserved homologies with human • Rich variety • Easily manipulated in gene expression	• Ethical requirements • High feeding and drug costs • Lack of typical clinical manifestations	(17, 18)
Rat	• Clinical symptoms similar to those in humans • Strong operability • Conserved homologies with human • Rich variety • Easily manipulated in gene expression	• Relative resistance to COPD development • Ethical requirements • High feeding and drug costs	(19, 20)
Guinea pig	• Similar anatomy and physiology to human lung	• Lack of molecular tools • High purchasing and raising costs • Ethical requirements	(21, 22)
Dog	• Respiratory pharmacology has been identified	• Large size with certain offensive • Ethical requirements • High feeding cost	(23)
Rhesus monkey	• Tracheal morphology closest to that of humans • Highly conserved genome in humans	• Ethical requirements	(24)
Miniature pig	• Lung volume similar to human	• Only induced by lipopolysaccharide	(25, 26)

COPD, chronic obstructive pulmonary disease.

In this paper, we summarize how *Drosophila* can model both cigarette smoke and non-cigarette smoke-induced tracheal inflammation. We evaluate the specific spectrum of phenotypic characteristics, immune responses, molecular mechanisms, and pathophysiological aspects accessible in *Drosophila* for interpreting COPD pathogenesis. Furthermore, the genetic features of *Drosophila* facilitate the screening of numerous candidate genes associated with phenotypes and the identification of effective drugs for COPD therapies. This suggests that the fly could serve as a powerful high-throughput platform for hypothesis generation and therapeutic target prioritization. By presenting a comprehensive and practical framework, we highlight *Drosophila* as a preclinical discovery tool capable of accelerating the screening process prior to validation in mammalian models and clinical trials.

## 2 Characteristics of *Drosophila* airway in COPD research

The *Drosophila* airway system has a simple structure with unique functions, making it an ideal biomedical model for exploring interconnected epithelial tubes and the branching morphogenesis of the respiratory system (27). More importantly, both fly tracheae and mammalian lungs share similarities in architecture and morphology, and they exhibit analogous physiological responses to various stimuli, such as smoke and pathogens (28, 29).

### 2.1 Airway homologies between *Drosophila* and human

**Figure 2:**
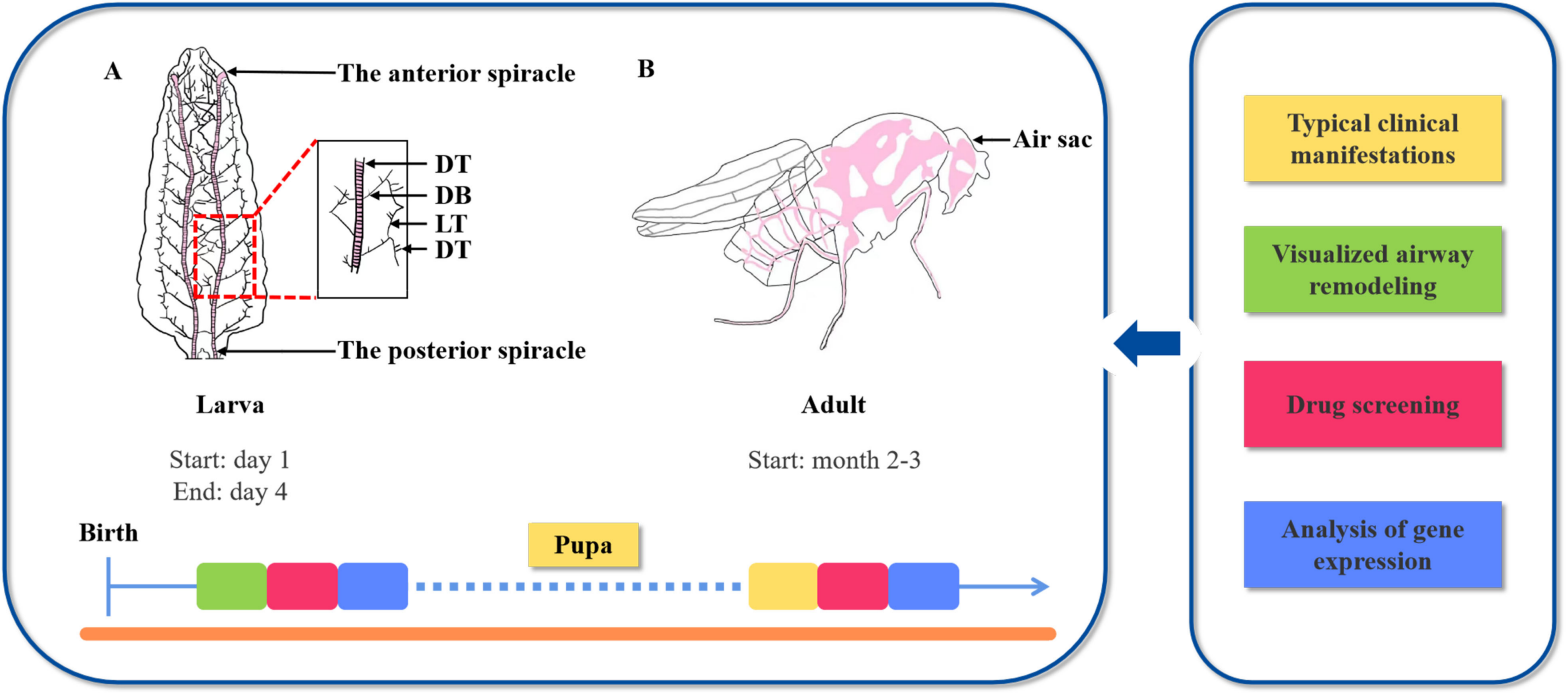
The schematic of *Drosophila melanogaster* airway system. **(A)** The airway of *Drosophila* can be divided into the following four characteristics: Type I branches, the dorsal trunk (DT) connects the open posterior spiracle and the anterior spiracle as the major airway; Type II branches, the lateral branch (LT) and dorsal branch (DB) as the primary branches; Type III tubes, the secondary branches; and Type IV, the terminal branches (TBs) as the larval terminal branches. **(B)** The difference in respiratory system underlying larvae and adults in *Drosophila*; tracheal system is shown in pink. During the metamorphosis stage, the simple structure of fly trachea evolves into a complex system; trachea in adult head and thorax develop into air sacs that center around the brain and flight muscles.

Flies undergo a four-stage life cycle: embryo, larva (comprising the first, second, and third instars), pupa, and adult. The larval airway system is bilaterally symmetrical and mainly consists of thousands of interconnected tubes (27). The formation of a single tracheal tube is based on the mechanism of airway epithelial morphogenesis, where epithelial cell layers surrounding a central lumen assemble into a tubule. This process is facilitated by the tight junctions between the epithelial cell layers, which ultimately shape the tube into an appropriate size for gas transport (30). Tracheal tubes transport oxygen and other gases through a network of tubules that includes primary, secondary, and terminal branches (31). Type I branches, such as the dorsal trunk (DT), serve as the major airway, connecting the open posterior spiracle to the anterior spiracle. Type II branches act as the primary branches and consist of the lateral branch (LT) and the dorsal branch (DB). Type III tubes represent the secondary branches, while Type IV tubes are the terminal branches (TBs) that get into the tissue cells (32) (**Figure 2A**). Functionally, fresh air (oxygen) diffuses from the spiracles into the terminal tubules and subsequently through the cell walls of the body, facilitating the gas exchange. In contrast, waste gas (carbon dioxide) diffuses in the opposite direction (32). Interestingly, there are significant differences in the airways among larvae, pupae, and adults. The major breathing branches in larvae exhibit a stereotypical and stable pattern, while the terminal tubules demonstrate high plasticity (33). The pupal and adult respiratory systems undergo extensive remodeling during the metamorphosis stage (34). Specifically, the network of larval tracheae transforms into air sacs in adults. These air sacs, located in the head and thorax of the fly, are positioned around the brain and flight muscles to facilitate gas exchange (35) (**Figure 2B**).

The airway system of flies shares similarities with mammals in both anatomical structure and physiological response. Anatomically, the larval tracheae resemble the bronchi in human respiratory organs, showing segmentation and bilateral symmetry (36, 37). In mammals, basal cells, ciliated cells, club cells, and goblet cells collectively defend against foreign substances in the airways (38). These cells secrete polypeptide mediators for initiating signal cascades and produce antimicrobial peptides (AMPs) to combat microbial invasions. AMPs work in conjunction with the mucus layer, forming a chemical barrier on the airway epithelium (39). In flies, the larval airway system consists solely of a single layer of epithelial cells. Consequently, the epithelium’s physiological responses to diverse stimuli occur independently of other cell types (40). Additionally, a chitinous inner lining fully spreads over the epithelial surfaces, maintaining tubular structure (37). More than 20 AMPs are synthesized on the epithelial surfaces to eliminate reactive oxygen species and combat airborne pathogen infections (41). Such AMPs include diptericin, drosocin, and attacin, which target gram-negative bacteria, while defensin mainly acts against gram-positive bacteria (42, 43).

### 2.2 *Drosophila* tracheal response to COPD

The fly airway system has evolutionarily developed appropriate mechanisms in response to various respiratory stresses, particularly in adapting to hypoxia and inflammation (44). Under hypoxic conditions, flies modulate mitochondrial functions by inhibiting the tricarboxylic acid (TCA) cycle metabolic pathway, which reduces oxygen consumption and maintains energy balance (45). This process is analogous to the hypoxia-inducible factor-1 alpha (HIF-1α) pathway in mammals, accounting for the dilation of the trachea and the improvement of gas exchange (46). Consistent with mammals, immune cells in the fly trachea have also presented an inherited mechanism, which is beneficial to facilitate trachea integrity. Crystal cells, a type of hemocyte, can transport oxygen through prophenol oxidase 2 protein (PPO2), helping to cope with respiratory stress during hypoxia (47). Furthermore, e-cigarette vapor has been used in adult fly models (48, 49). During development, nicotine insult leads to an increase in the brain hemisphere size in larvae and a decrease in the number of tyrosine hydroxylase-positive (TH+) neurons in the adult dopaminergic system (50). These methodologies make *Drosophila* an appropriate model for interpreting the evolutionary mechanisms of COPD.

Although there are anatomical differences between the tracheal systems of flies and humans, the fly trachea is practical for exploring airway dysfunctions in COPD. Cigarette smoke exposure can effectively establish a COPD model in *Drosophila*, replicating representative manifestations observed in COPD patients, including hypoxia, tracheal remodeling, shortened lifespan, increased metabolic rates, and loss of body fat (51). COPD severity is closely associated with bronchial disruptions (52). Consistent with the progressive deterioration of tracheal morphology seen in mammals, flies exhibit a decrease in respiratory epithelial surface area, a reduction in the number of terminal tracheae, shortening of tracheal length, and even epithelial cell hyperplasia (37, 53, 54). Additionally, the heart rates in both larvae and adults are elevated due to alterations in the dynamic variation of intracellular calcium in myocardial cells caused by cigarette smoke exposure (55). Taken together, *Drosophila* is a viable model to validate the implicated pathogenesis of COPD and to reproduce specific pathophysiological symptoms.

### 2.3 Cigarette smoke induction of COPD in the fly model

**Figure 3:**
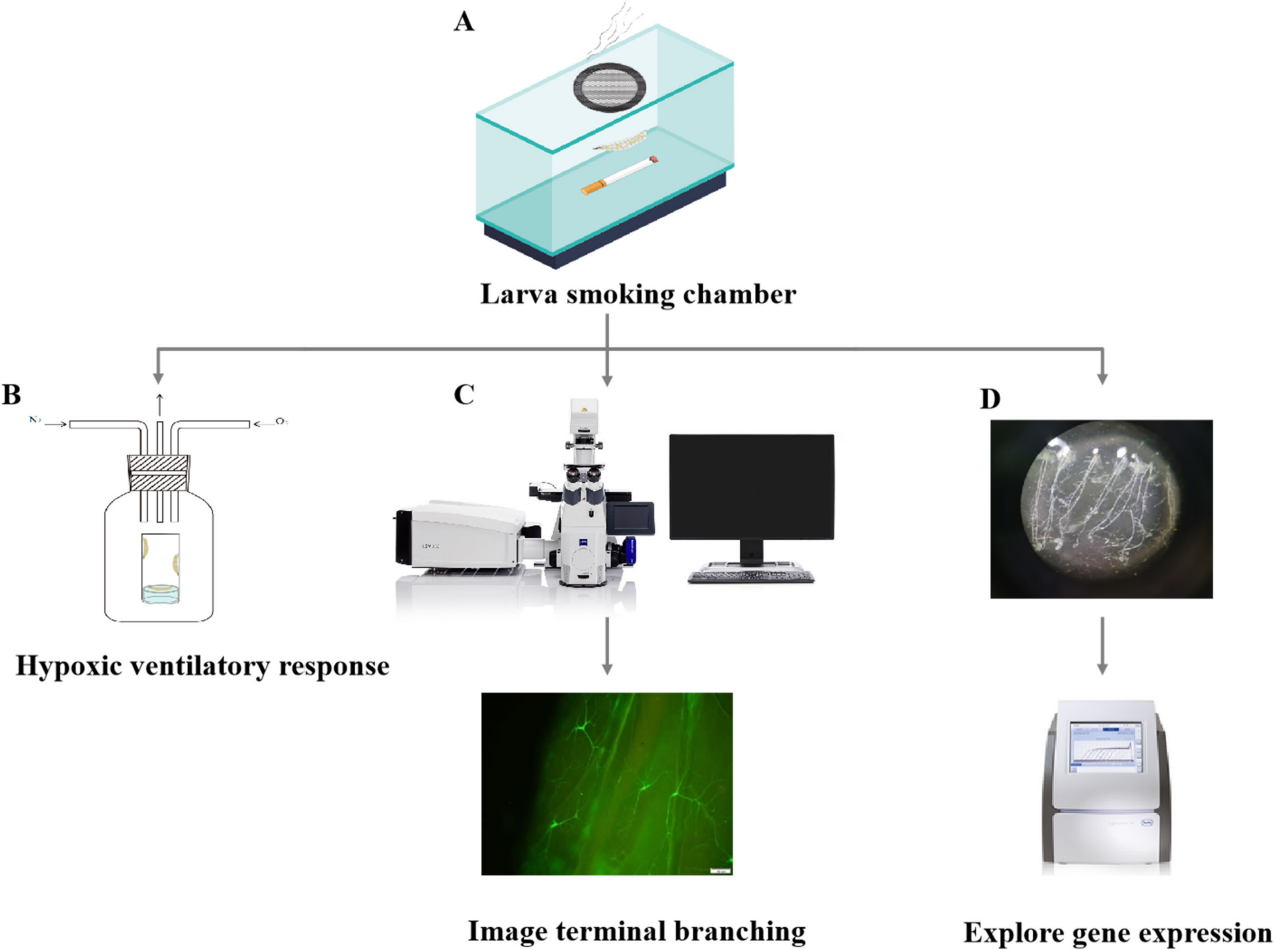
Indicators of cigarette smoke exposure in larvae. **(A)** Larvae are collected and transferred into the smoking chamber, where they are exposed to cigarette smoke. **(B)** Vial is filled with oxygen, and nitrogen gas is used for testing hypoxia sensitivity. **(C)** Airway branching morphologies under a confocal microscope are analyzed. **(D)** Tracheae of cigarette smoke-induced larvae are dissected and undergo gene expression test.

**Figure 4:**
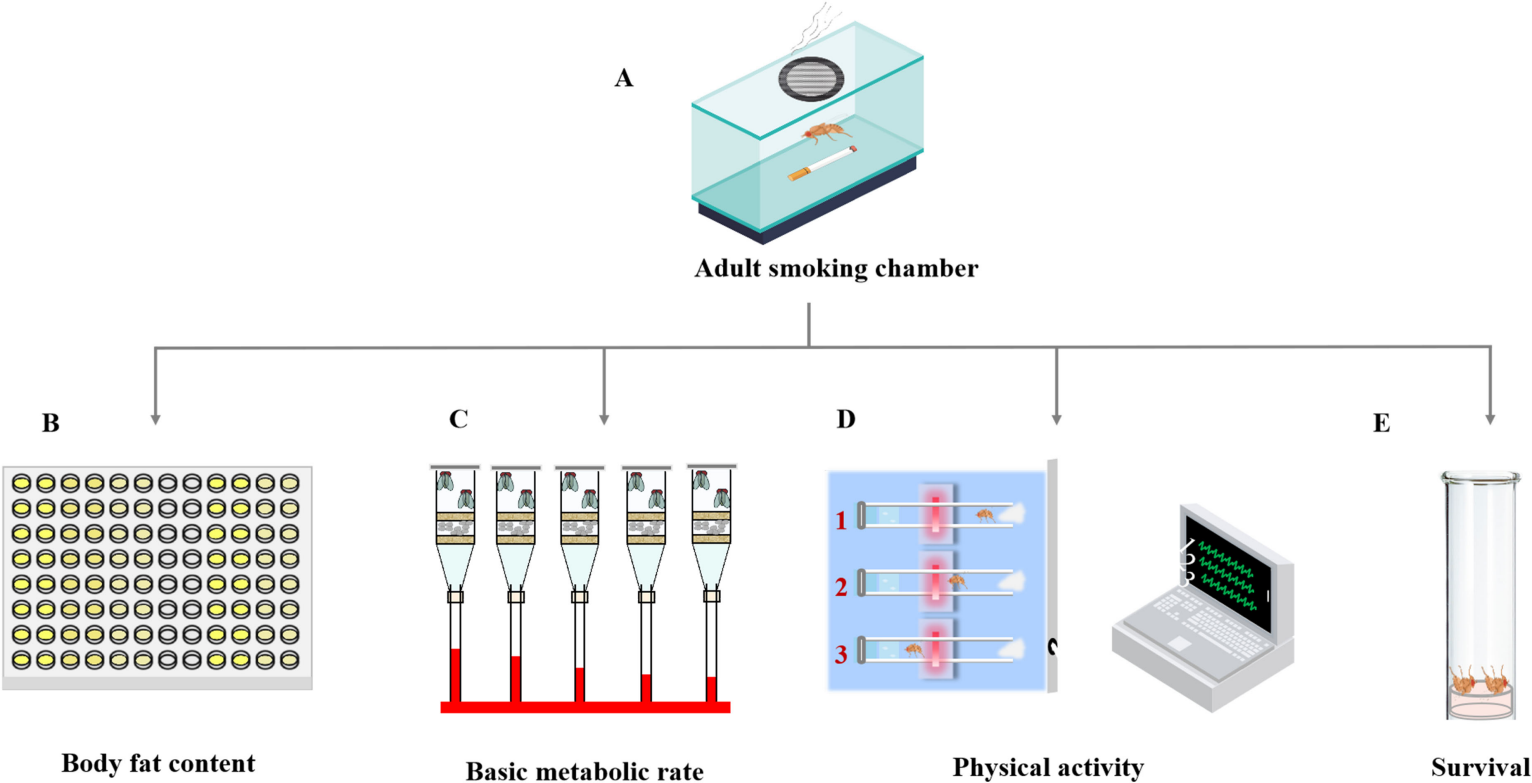
Indicators of cigarette smoke exposure in adults. **(A)** Adults are collected and transferred into the smoking chamber, where they are exposed to cigarette smoke. **(B)** The body fat content is measured using ELISA. **(C)** The basic metabolic rate is quantified by the volume of CO_2_ production. **(D)** The declination of the physical activity is monitored using the DAM system. **(E)** The lifespan measurement is applied by counting dead flies. DAM, *Drosophila* Activity Monitoring.

Cigarette smoke exposure is the primary method for modeling COPD in rodent studies, while similar smoking regimens have been adopted in fly models (56, 57). Flies undergo metamorphosis, and their airway structures change distinctly throughout development. Although both larval and adult models involve inhaling smoke, they differ in specific details. In the larval model, second-instar larvae are collected and transferred into a smoking chamber, where they are exposed to cigarette smoke three times a day for 30 minutes over 2 days (**Figure 3A**) (56). In the adult model, 5- to 7-day-old adult flies are exposed to cigarette smoke for 30 minutes each day over 5 days (**Figure 4A**) (56).

In addition to the differences in modeling, the two types of models also employ various approaches to interpret pathophysiological indicators of COPD. In brief, the larval model is primarily used to investigate molecular cascades, tracheal morphology, and behavioral changes associated with COPD (56, 58). Anoxia, a common clinical symptom in COPD patients, is observed in cigarette smoke-exposed larvae. The larvae are transferred into vials containing 2.5% to 4% oxygen and nitric oxide to monitor larval intolerance to hypoxia and their ventilatory response to oxygen deficiency (**Figure 3B**). Airway remodeling, another pathological change seen in COPD patients, is also observed in larvae after cigarette smoke exposure. Currently, the *dsrf*-Gal4; UAS-*gfp* flies can be utilized to analyze terminal branching morphologies following cigarette smoke exposure. The dorsal branches of the fly airway system are analyzed using NeuronJ (**Figure 3C**). To explore gene expression in the trachea of cigarette smoke-exposed larvae, third-instar larvae are dissected in cold Phosphate-Buffered Saline (PBS). Subsequently, the dissected trachea is transferred into RNA TRIzol Reagent for quantitative reverse transcription polymerase chain reaction (qRT-PCR) experiments (**Figure 3D**) (56, 58).

Concurrently, the cigarette smoke-induced adult model has revealed a complex array of systemic symptoms, including emaciation, premature death, and exercise intolerance (56, 57). These symptoms are also observed in COPD patients. Briefly, body fat content is measured using an enzyme-linked immunosorbent assay (ELISA) (**Figure 4B**), basal metabolic rate is quantified by measuring the volume of carbon dioxide (CO_2_) production (**Figure 4C**), the physical activity is monitored using the *Drosophila* Activity Monitoring (DAM) system (**Figure 4D**) (56), and survival rate is determined by counting the number of survivors daily (**Figure 4E**) (56, 58). Overall, these methods are straightforward to implement for establishing a COPD *Drosophila* model.

### 2.4 Non-cigarette smoke induction of COPD in the fly model

COPD is a heterogeneous condition triggered by multiple contributing factors. Inhalation of air pollutants, such as pollutant gases and particulate matter (PM), is a significant causative factor in both the initiation and exacerbation of COPD (59). PM refers to particles suspended in the air that consist of solid or liquid components, typically with diameters less than 10 μm. Mechanistically, PM are directly inhaled and deposited deep within the lungs, and their xenobiotic properties play a decisive role in promoting lung inflammation (59). Given the benefits of flies, PM exposure can effectively transform *Drosophila* into a consequential COPD model. The first is modeling methods; strategies employed in PM models include intratracheal instillation, intranasal instillation, mouth-nose inhalation, and whole-body exposure chambers (60). Among these, the inhalation of PM and whole-body exposure chambers are applicable to flies, as this mode of entry is comparable to cigarette smoke exposure in *Drosophila*. Second, the typical pathophysiological changes underlying COPD, such as small airway remodeling and inflammation, have been induced by PM exposure; these pathological features can also be replicated in established fly models (61). Third, the pathogenesis associated with PM exposure encompasses key mechanisms including cytokine release, mitochondrial dysfunction, impaired immune function, oxidative stress imbalance, and the over-activation of inflammatory pathways (60, 62). Finally, the genetic tractability of *Drosophila* offers extensive opportunities to identify potential genes and screen therapeutic candidates for non-cigarette smoke-induced COPD.

### 2.5 Innate immune system of *Drosophila* airway

Numerous studies have highlighted the prominent role of innate immune responses to COPD. Massive research conducted on smoking mice indicates that innate immunity plays a leading role in driving COPD progression, particularly during the early phases of pulmonary changes (63, 64). Furthermore, the innate immune system even works longer than the adaptive immune system throughout the course of COPD (65). However, murine models present challenges in elucidating the innate immune mechanisms involved in COPD development due to the complex interrelations between innate and adaptive immunity (66).

*Drosophila* has worked as an ideal model for elucidating the mechanisms of innate immunity in human diseases because it possesses only innate immunity and lacks adaptive immunity (67, 68). Although fly models cannot fully represent COPD mechanisms that are largely influenced by antigen-specific lymphocytes—such as adaptive immune amplification, lymphoid aggregate or follicle-like features, and immune memory—*Drosophila* possesses a conserved innate immune system that can be used to investigate epithelial stress responses and exacerbations, as well as to initiate and sustain inflammatory remodeling procession (69). Consequently, the fly model is well-suited to facilitate the analysis of the relationship between innate immunity, COPD, and inflammation (70, 71). Furthermore, immune-related findings from flies must subsequently be validated in mammalian systems.

## 3 Conserved COPD signaling pathways between *Drosophila* and vertebrates

**Figure 5:**
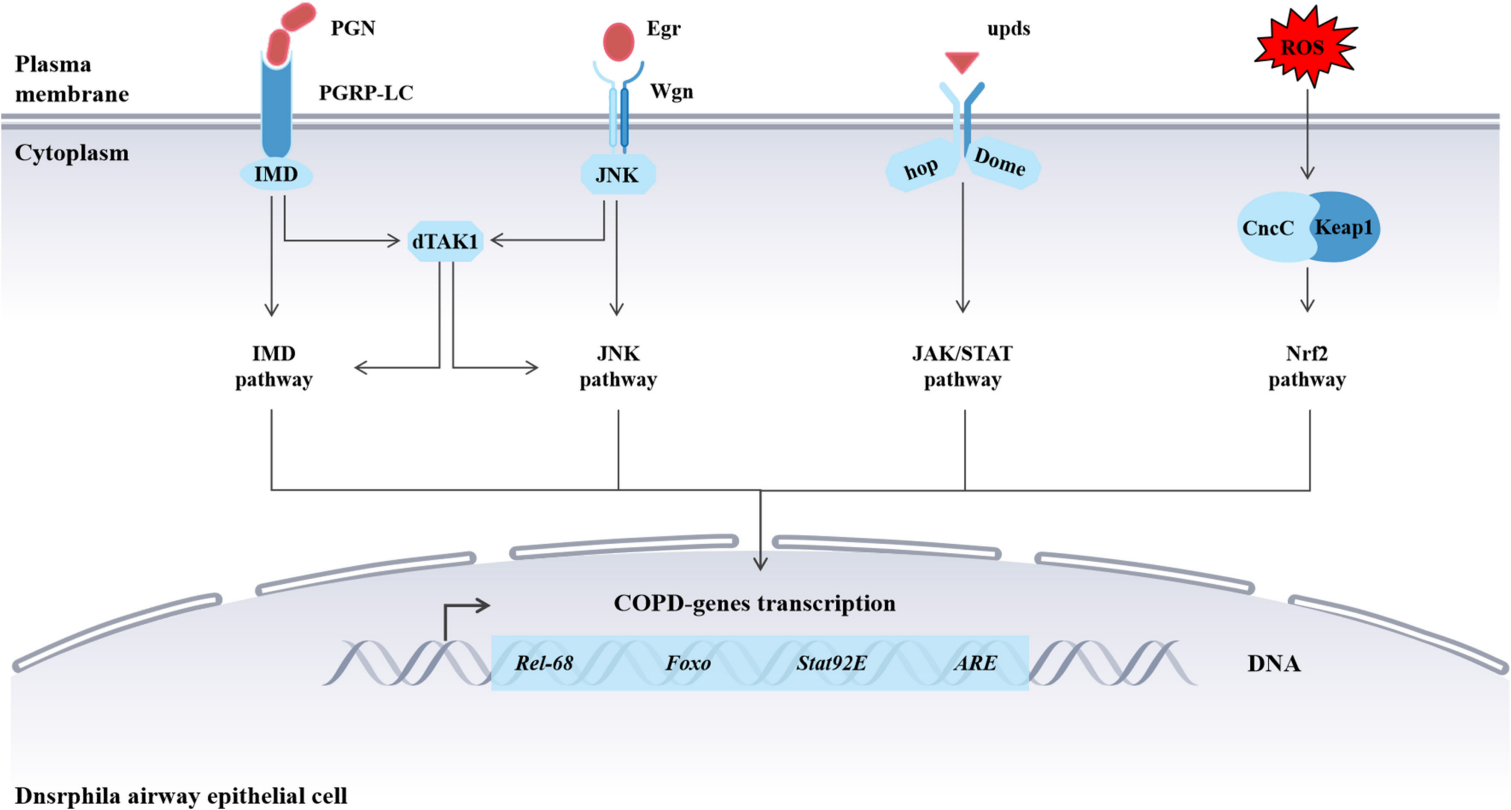
Molecular mechanisms in *Drosophila* model respond to cigarette smoke interference. Signaling pathways in *Drosophila* model respond to cigarette smoke interference, including IMD, JNK, Nrf2, and JAK signaling. Specifically, the mechanisms and functions of these pathways are similar to those in mammals.

COPD exacerbations are inextricably related to chronic inflammation, characterized by the activation of signaling pathways, increased immune cell activity, and elevated levels of inflammatory mediators in the respiratory tract (72, 73). Cigarette smoke can also initiate airway epithelial inflammation in flies, leading to the activation of signaling pathways, the generation of AMPs, and a significant increase in inflammatory mediators, similar to the response observed in mammals (71, 74). This section summarizes some key signaling pathways in *Drosophila* during COPD progression (**Figure 5**) and provides a simplified comparison (**Table 2**) of the mechanistic details between flies and mammals for these pathways.

**Table 2:** Comparative analysis of COPD-related signaling pathways between *Drosophila* and mammals.

Homologous elements	NF-kB pathway		References
	*Drosophila melanogaster*	Mammal	
Pathway name	• Toll pathway • IMD pathway		(42, 75)
Activated signal	PGNs	PAMP, cytokines (TNF-α), external stimulus	(75–79)
Receptor	Toll, PGRP-LC	PRRs, TLR, TNFR	(78, 79)
Transcription factor	• Toll: Dorsal, Dif • IMD: Rel	p50–p65, p52-RelB	(78–80)
Signaling cascade	• IMD: PGNs → PGRPs → adaptor proteins → IκB complex → Dredd caspase cleavage Rel → N-terminal entry nuclear • Toll: several elements are missing in the tracheal system.	Ligands → receptors → IκB complex → IκBα degradation → p50–p65 into the nucleus	(76, 79–82)
Biological function	• Anti-infections • Embryonic development	• Immunity and inflammation • Cell survival/proliferation • Development • Stress responses • Tumor progression	(75, 77, 78, 83–87)
Response to COPD	• Epithelial cell proliferation • Produce AMPs • Eliminate pathogens	• Epithelial cell proliferation • Activate airway inflammation • Anti-apoptosis • Eliminate pathogens	(76, 79, 80, 82, 83)
Homologous elements	JAK/STAT pathway		References
	*Drosophila melanogaster*	Mammal	
Activated signal	Only upd, upd2, upd3 (IL-6 homolog)	Multiple cytokines/hormones	(79, 88, 89)
Receptor	Dome (IL-6R homolog)	Multiple receptors	(79, 88, 90)
JAK kinase	Hop (JAK2 homolog)	JAK1, JAK2, JAK3, and TYK2	(79, 88, 90)
STAT transcription factor	Stat92E (STAT5 homolog)	STAT1, STAT2, STAT3, STAT4, STAT5A, STAT5B, and STAT6	(79, 88, 91, 92)
Biological function	• Embryonic development • Regulate immune response • Tissue repair • Determine gender	• Regulate immune response • Mediate apoptosis • Tissue repair • Growth, development, and metabolism • Tumor progression	(92–95)
Response to COPD	• Narrow the tracheal lumen • Lead to trachea epithelium hypertrophy • Mediate AMPs	• Lead to pulmonary fibrosis • Amplify airway inflammation	(58, 89, 95–98)
References	JNK pathway		Homologous elements
	*Drosophila melanogaster*	Mammal	
MAP3K	Tak1, Slpr, Msn	ASK1, MEKK1/4, MLK families	(71, 99)
MAP2K	Hem	MKK4, MKK7	(71, 99)
MAPK	Bsk	JNK1, JNK2, JNK3	(71, 99)
Activated signal	• Development signals: epidermal growth factor (EGF) • Cytokines: Eiger (TNF homolog) and Mmp1/Mmp2 • Stress signals: oxidative stress	• Stress signals: oxidative stress, DNA damage, ultraviolet (UV) • Inflammatory factors • Growth factors	(71, 99–104)
Receptor	Wgn (TNFR homolog)	Multiple receptors	(71, 99)
Signaling cascade	MAP3K → Hem → Bsk	MAP3K → MKK4/7 → JNK	(71, 99)
Transcription factor	AP-1 complex (dJun)	AP-1 complex (c-Jun)	(71, 99)
Biological function	• Regulate immune response • Embryonic development • Response to stress • Induce apoptosis • Tissue repair	• Tissue repair • Regulate inflammatory response • Induce apoptosis • Response to stress • Dual responses: initiate protective/damaging response to cells	(71, 74, 99, 102, 105)
Response to COPD	• Amplify airway inflammation • Promote remodeling in larval airway	• Promote remodeling in airway and alveolar • Promote airway mucus hypersecretion • Amplify airway inflammation	(74, 106–110)
Homologous elements	Nrf2 pathway		References
	*Drosophila melanogaster*	Mammal	
Nrf2 homolog	CncC	Nrf2	(111–113)
Keap1 homolog	dKeap1	Keap1	(111–113)
Activated signal	• Oxidative stress • Electrophiles • Certain xenobiotics	• Oxidative stress • Electrophiles • Multiple xenobiotics	(111–113)
DNA binding sequence	AREs	AREs	(111–113)
Activated mechanism	The dKeap1 is modified by the stressors, resulting in conformational changes. This dKeap1 is unable to degrade CncC. CncC is accumulated and then incorporated into the nucleus to bind AREs, leading to antioxidant gene expression.	The mammal Nrf2 mechanism is exactly the same as that of flies.	(114, 115)
Biological function	• Anti-oxidative stress • Detoxification • Anti-inflammation • Prolong lifespan and aging • Development	• Anti-oxidative stress • Detoxification • Regulate metabolism • Anti-inflammation • Prolong lifespan and aging	(116–119)
Response to COPD	• Alleviate trachea inflammation • Produce antioxidant enzymes	• Alleviate COPD aggravations • Produce antioxidant enzymes	(56, 113, 115, 117, 118, 120, 121)

COPD, chronic obstructive pulmonary disease; IMD, immune deficiency; PGNs, peptidoglycans; PAMP, pathogen-associated molecular pattern; PGRP, peptidoglycan recognition protein; PRRs, pattern recognition receptors; TLR, Toll-like receptor; TNFR, tumor necrosis factor receptor; AMPs, antimicrobial peptides; JAK/STAT, Janus kinase/signal transducer and activator of transcription; AREs, antioxidant response elements.

### 3.1 NF-κB pathway

Nuclear factor-kappa B (NF-κB) is a key mediator of inflammatory cascades and is involved in development, tumorigenesis, inflammation, and immune responses (75). The NF-κB pathway can be activated by a variety of factors, including pathogens, cytokines, and physical and chemical stimuli. Dysregulation of NF-κB signaling can cause a variety of diseases, such as cancer, atherosclerosis, arthritis, and immunological disorders (76). NF-κB also plays a crucial role in several pulmonary pathologies, including COPD (76). The respiratory epithelium, when exposed to cigarette smoke or other noxious stimuli, can secrete numerous pro-inflammatory cytokines, one of which is tumor necrosis factor-alpha (TNF-α). TNF-α binding to its receptor can initiate the NF-κB signaling pathway (78). Consequently, the elevation of TNF-α levels and the over-activation of NF-κB lead to the pathology of COPD. Pathogen-associated molecular patterns (PAMPs) can be recognized by pattern recognition receptors (PRRs), such as Toll-like receptors (TLRs), causing the translocation of NF-κB into the nucleus. The Toll pathway has been identified as a molecular mechanism involved in the pathogenesis of COPD, with the primary functions of eliminating invading pathogens and initiating inflammatory responses (79, 80).

The NF-κB pathway is essential for *Drosophila* development and host defense, including dorsal and ventral axis polarity, infection resistance, and innate immune homeostasis (82, 83). The immune deficiency (IMD) pathway in *Drosophila* shares evolutionarily conserved signaling molecules with the TNF-α pathway in mammals. Most importantly, the activation of the IMD pathway requires members of the NF-κB transcription factor family (122). All genes corresponding to the canonical components of the IMD signaling cascade are expressed in the fly airway system (79). The IMD pathway converges on the transcription of corresponding antimicrobial peptide genes, which are crucial for maintaining epithelial integrity as flies encounter noxious particles (74). The first step in IMD signal transduction is that peptidoglycan recognition proteins (PGRPs) interact with peptidoglycans (PGNs). Upon initiation by an inflammatory inducer, the IMD pathway regulates the activation of a complex composed of IMD, Fadd, and Dredd. The caspase Dredd cleaves the inhibitory C-terminal region of a third *Drosophila* NF-κB transcription factor known as Relish (Rel) and also cleaves the adaptor protein IMD, leading to the recruitment and activation of the transforming growth factor-β-activated kinase 1/TAK1-binding protein 2 (Tab2/Tak1) complex. Upon phosphorylation, the Tab2/Tak1 complex activates the IκB kinase (IκB) complex. Subsequently, the IκB complex induces the phosphorylation of multiple sites on the N-terminal region of Rel. The active N-terminal fragment (Rel-68) translocates into the nucleus to promote AMP expression (84, 123). AMP expression in the airway epithelium is primarily driven by the IMD signaling pathway, which is further modulated by other pathways such as Janus kinase (JAK), c-Jun N-terminal kinase (JNK), and the Toll pathway (85). Recent discoveries have shown that cigarette smoke exposure in *Drosophila* activates the IMD signaling pathway in the respiratory tract epithelium, resulting in the increased expression of antimicrobial peptides and the thickening of the airway tubes (56, 74).

**Figure 6:**
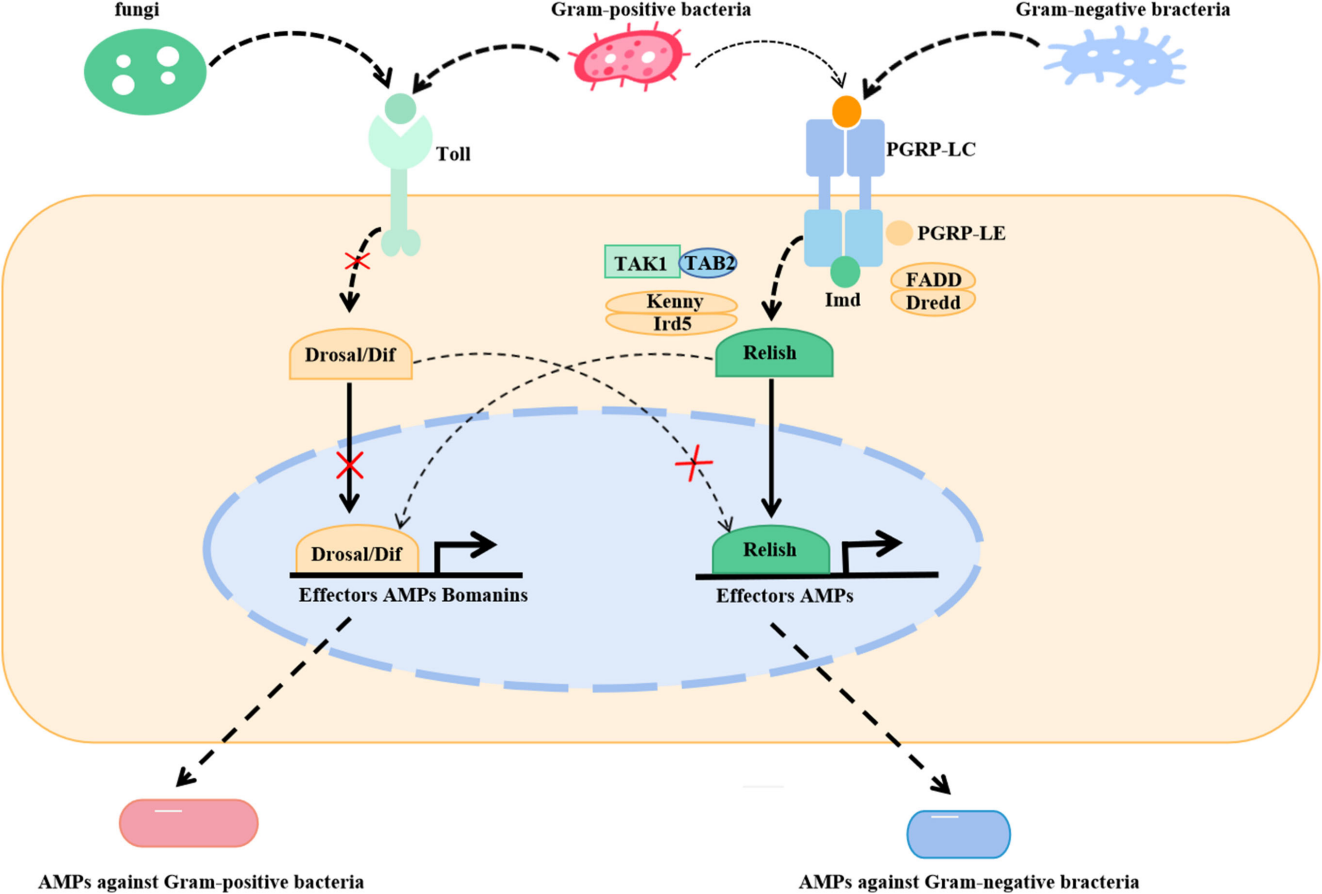
Antimicrobial peptides are mediated by Toll and IMD signaling pathways in airway epithelial cells. Toll pathway interacts with fungi and gram-positive bacteria, and IMD pathway interacts with gram-positive bacteria and gram-negative bacteria. Upon the airway epithelium, a portion of Toll pathway effectors is absent, but all members required for proper function of IMD pathway are expressed; both of them can attract NF-κB family members. Therefore, the cross between Toll and IMD pathways may adjust and control AMPs, as shown in the figure. IMD, immune deficiency; AMPs, antimicrobial peptides.

The Toll pathway in *Drosophila* takes a central position in defending against fungal and gram-positive bacterial invasions (86). Notably, key components of this cascade are evolutionarily conserved in mammals (80). However, the Toll pathway is defective in the fly airway system; among the most important causes is that the intracytoplasmic components of the entire Toll complex, such as Tube and Pelle, are deficient, while membrane-bound receptors and nuclear transcription factors remain intact (79). Despite these deficiencies, the pathway can still mediate AMPs against infections, as both the Toll and IMD pathways can recruit members of the NF-κB family. The crosstalk and interplay between these two pathways may provide a proper mechanism to maintain homeostasis in the airway epithelium (**Figure 6**) (42, 87). Therefore, *Drosophila* serves as an ideal model for studying the NF-κB signaling pathway associated with COPD.

### 3.2 JAK pathway

The JAK/signal transducer and activator of transcription (JAK/STAT) pathway interacts with a great diversity of cytokines to modulate physiological functions. It is characterized by the transduction of extracellular signals into the nucleus, where it regulates the expression of specific genes (89). The JAK/STAT pathway is involved in immune responses, tissue repair, apoptosis, and cell proliferation (93). Dysfunction of the JAK/STAT pathway has been related to various diseases, including COPD, cancer, polycythemia, and gigantism (90, 96). More than 50 cytokines and growth factors, such as interferons (IFNs), TNF, and interleukins (ILs), can activate the JAK/STAT pathway in mammalian cells (89). The activation of the JAK/STAT pathway is implicated in the onset and exacerbation of COPD (96). Recent studies have demonstrated that specific JAK inhibitors can clinically improve pulmonary fibrosis and reduce neutrophil activation during COPD progression (91, 97).

The *Drosophila* JAK/STAT pathway is critical in regulating various biological processes, including organ development, physiological homeostasis, and immune responses (88, 92). In mammals, the molecular components of the JAK/STAT pathway consist of four Janus kinases (JAK1, JAK2, JAK3, and TYK2) and seven transcription factors (STAT1, STAT2, STAT3, STAT4, STAT5A, STAT5B, and STAT6) (90). By contrast, *Drosophila* possesses only a single kinase, hopscotch (hop), which is homologous to human JAK2; a single receptor, Domeless (Dome), homologous to human IL-6R; and a single transcription factor, STAT92E, homologous to human STAT5 (92). The corresponding ligands of JAK/STAT signaling, such as unpaired (upd), upd2, and upd3, are the homologs of IL, with upd3 being particularly conserved with IL-6 (124). Although the JAK/STAT pathway is less complex in *Drosophila*, all core elements of this cascade are expressed in the tracheal system (79). JAK/STAT signaling not only is of great importance during tracheal development but also plays a vital role in maintaining the integrity of the airway epithelium (94). Mechanistically, cigarette smoke induces excessive activation of the JAK/STAT signaling pathway and increases the levels of upd2 and upd3 (95). The expression of upd3 is induced by the substantial accumulation of reactive oxygen species (ROS) in the respiratory system (98). When upd2 and upd3 bind to the extracellular domain of the Dome receptor, Dome dimerizes to transmit signals into the intracellular domain. This signaling cascade ultimately induces the expression of epithelial cell repair genes, leading to significant structural changes that disrupt normal respiratory functions (95). Specifically, the JAK/STAT pathway triggers hypertrophy of the tracheal epithelium in larvae, resulting in excessive thickening that narrows the lumen and substantially disrupts the tracheal epicuticular architecture (58, 95). In addition, the JAK/STAT pathway mediates the expression of antimicrobial peptides in response to airway inflammation (58).

### 3.3 JNK pathway

The JNK, commonly known as the stress-activated protein kinase (SAPK), represents a subgroup of the mitogen-activated protein kinase (MAPK) family. Multiple stressors are integrated into the classical JNK cellular signaling cascade through MAP3Ks, MAP2Ks, and MAPKs, which comprise the JNK pathway, resulting in either protective or damaging responses (100). Under normal conditions, the JNK pathway maintains homeostasis and barrier function in the human airway epithelium (99). Contrastingly, under cigarette smoke exposure, the JNK pathway undergoes prolonged activation and is markedly upregulated (107). Sustained activation of the JNK pathway can drive various forms of cell death, including apoptosis, necrosis, and autophagy (108). The role of JNK in the pathophysiology of COPD involves three key aspects: it promotes the remodeling of both small airways and alveolar walls, contributing to decreased airway elasticity and reduced lung compliance during COPD exacerbations (31, 48, 50); it mediates airway mucus hypersecretion, leading to airway obstruction during acute COPD exacerbations (56, 88); and it further aggravates the inflammatory response of the airway epithelium during COPD development (43, 48).

The *Drosophila* JNK signaling pathway is crucial for various biological activities, including embryonic development, immune response, stress response, cell migration and differentiation, and apoptosis (101, 102, 125). JNK signaling is a highly conserved pathway between mammals and *Drosophila* (101). Unlike humans, which have three JNK genes, *Drosophila* possesses only a single JNK gene, known as *Basket (bsk)* (125). Several proteins are effective in the *Drosophila* JNK pathway; notable effectors include Eiger (Egr) and matrix metalloproteinases 1 and 2 (Mmp1 and Mmp2). Egr can activate the JNK pathway through its receptor Wengen (Wgn) to regulate biological activities in the airway, such as melanization (103). Egr is homologous to the TNF family, while Wgn is homologous to the TNF receptor. Mmp1 and Mmp2 are modulated by the JNK pathway and are indispensable components for tissue remodeling in *Drosophila*; they are the congener of human MMP-2 and MMP-9, which have been characterized as key editors of lung fibrosis in COPD patients (109, 126, 127). In addition to Egr and Mmps, JNK signaling induces epithelial apoptosis in both mammals and flies (101, 110). The JNK and IMD pathways cooperatively regulate tube morphology following the chronic inflammation of the epithelium, resulting in increased thickness of airway walls (74). Cigarette smoke exposure can elevate the levels of upd in flies and enhance the activity of the JNK pathway and ligands of the JAK/STAT pathway (56). Consequently, the JNK pathway may cooperate with the JAK/STAT pathway to regulate tube morphology (71, 105). The *Drosophila* JNK pathway exhibits temporal modulation characteristics. In the context of COPD, epithelial cells are continuously stimulated by oxidative stress and inflammatory cytokines, leading to amplified inflammation and the remodeling of the trachea in *Drosophila* larvae. Although the JNK signaling pathway is a complex, interconnected pathway, its relative simplicity in *Drosophila* provides valuable insights into the etiology of COPD and can be utilized as a platform for exploring novel therapeutic agents.

### 3.4 Nrf2 pathway

Nuclear factor erythroid-2-related factor 2 (Nrf2), a member of the Cap ‘n’ collar-basic region leucine zipper (CNC-bZIP) family of transcription factors, is a predominantly principal regulator of cellular defense mechanisms. It encodes the expression of antioxidant and detoxification genes that protect against oxidative stress, inflammatory injury, carcinogenesis, and physiological metabolism (128). Multiple oxidative and chemical stresses can reduce Nrf2 expression in the respiratory tract, contributing to a range of pulmonary diseases, including COPD, idiopathic pulmonary fibrosis (IPF), acute respiratory distress syndrome (ARDS), and lung carcinoma (113). Under normal cellular conditions, Nrf2 is inhibited by the endogenous repressor Kelch-like ECH-associated protein 1 (Keap1) and is targeted for proteasomal degradation in the cytoplasm (113). Upon activation, Nrf2 translocates to the nucleus and binds to antioxidant response elements (AREs) to counteract endogenous oxidative stress (115). Upon cigarette smoke exposure, the Nrf2 signaling pathway is significantly activated; however, the progression of COPD in smokers is inversely correlated with Nrf2 pathway activation (117). Among the pathological factors exacerbating COPD, oxidative stress is considered a primary contributor resulting from cigarette smoke exposure (118). Cigarette smoke causally induces the accumulation of numerous endogenous oxidants (e.g., nitrogen, carbon, and particulate matter) and exogenous oxidants (e.g., superoxide, hydrogen peroxide, and hydroxyl radicals) to accumulate in lung tissues, leading to a redox imbalance in the airways and impaired Nrf2 regulation (120). Nrf2 agonists represent a promising therapeutic strategy for mitigating COPD aggravations (121).

The *Drosophila* Nrf2 pathway is functionally conserved with that of mammals (114). In flies, the Nrf2 pathway is involved in a variety of biological processes, including mediating embryonic development, extending lifespan, maintaining intestinal stem cell homeostasis, resisting oxidative stress, and enhancing xenobiotic metabolism (129–131). *Drosophila* possesses three Cap ‘n’ collar (Cnc) proteins, designated as CncA, CncB, and CncC. Among these, CncC functions as a stress regulator that closely resembles mammalian Nrf2 (114). In addition to CncC, other canonical and core elements of the Nrf2 pathway, such as Keap1 and AREs, are also evolutionarily conserved and orthologous to their vertebrate counterparts (114). The Nrf2 pathway in the *Drosophila* airway system is proposed to be a significant regulator against elevated levels of xenobiotics and ROS induced by cigarette smoke exposure (31, 56, 132). Under cigarette smoke stimulation, the fly respiratory tract generates excessive oxidative stress and inflammatory mediators, which activate Nrf2 to drive the transcription of target genes related to antioxidant enzymes and cytoprotective proteins (31). Recent studies have revealed that Nrf2 signaling in the fly airway system is significantly upregulated following cigarette smoke exposure (31, 56). In addition to antioxidants, the expression of genes involved in oxidative stress responses is also markedly increased (31, 56). Therefore, the Nrf2 pathway in *Drosophila* serves as a valuable model for analyzing the balance between oxidative and antioxidant mechanisms in the pathogenesis of COPD.

## 4 *Drosophila* as a genetic tool in COPD research

Functional genes can be identified and analyzed using advanced methodologies. These corresponding genes can then be leveraged for mechanistic studies and drug development (51, 133). *Drosophila* can work as a genetic tool for COPD investigation, including studying gene function, developing treatments, and conducting pharmacological research (134).

### 4.1 Studying genes associated with COPD in *Drosophila*

High-throughput genetic screening (HTGS) has been widely used to analyze gene functions, including the discovery of disease-related genes, and the manipulation of gene expression (135). Currently, HTGS is available for studying genes implicated in the COPD phenotype (136, 137). *Drosophila* offers unique advantages for the genetic dissection of COPD-related genes (138). i) Its short life cycle and robust reproductive capability facilitate genetic crosses and the establishment of transgenic lines. ii) Its ease of feeding and small size make it well-suited for HTGS. iii) Various phenotypes, including morphology, behavior, and physiology, can be easily measured (56). iv) Its genome is relatively simple, and whole-genome sequencing has been completed (139, 140). v) Multifarious factors related to COPD are evolutionarily conserved from mammals to flies, including genes, molecular mechanisms, and symptoms (31, 51). Specifically, the susceptibility genes have been identified through genome-wide association studies (GWASs) for COPD; these genes contribute to personalized disease manifestations, including 156 genes associated with 82 decisive COPD loci (141). Fly models have been applied to identify such susceptibility genes, including human ortholog genes CHRNA3 and CSMD1, which are involved in alpha-1 antitrypsin deficiency (AATD), a key factor in COPD pathogenesis (142). vi) Efficient gene-editing techniques in flies, such as CRISPR/Cas, GAL4/UAS, FLP/FRT, LexA-LexAop, QF-QUAS, and Cre/loxP systems, have been exploited to manipulate gene expression (143–145). For example, the GAL4/UAS gene expression system, a powerful genetic tool, is highly effective for investigating COPD-associated symptoms (146, 147). Serum response factor (Dsrf), a specific marker for terminal tracheal cells, can be utilized to produce *dsrf*-Gal4; UAS-6x *gfp* reporter flies, in which the terminal tubes are marked with Green Fluorescent Protein (GFP), facilitating the assessment of tracheal alterations following cigarette smoke exposure. *Prx2540*-GAL4; UAS-*gfp* and *ARE*-GAL4; UAS-*gfp* flies have been employed to verify the role of the Nrf2 signaling pathway in combating oxidative stress during COPD (56). vii) The simple organizational structure and the transparent body of flies allow researchers to rapidly observe and analyze their characteristics. Therefore, *Drosophila* is a potent tool for identifying functional genes involved in COPD.

### 4.2 Screening targets in COPD therapies by *Drosophila*

Both synthetic and natural chemical libraries have recently expanded in modern medical research, necessitating the development of novel model organisms to screen active ingredients from numerous compounds. The *Drosophila* model appears more properly in high-throughput screening (HTS) against targets compared to rodent models (**Table 3**) (57, 148). First, prolonging life is of paramount importance for COPD patients. Survival experiments can be effectively conducted in flies rather than mice, alleviating ethical concerns (149, 150). Second, the use of targeted GFP allows the visualization of tracheal alterations in the COPD fly model without invasive procedures during pharmacological interventions (56, 151). Third, therapeutic drugs effective in human pulmonary cells act with parallel efficacy in flies (152, 153). Fourth, drug administration methods in flies are diverse, including permeabilization, oral administration, vapor diffusion, and abdominal injection (57). These approaches facilitate the exploration of the pharmacodynamics and pharmacokinetics of COPD therapeutics.

**Table 3:** Relative advantages and disadvantages of *Drosophila* and mice for drug discovery.

Model	Advantages	Disadvantages	References
*Drosophila*	• Short life cycle • An innate immune model for investigating diseases • A large number of homologs with human • Various strains for high-throughput screening • Multiple modes of administrations • Easy to handle • Suit for probing pharmacokinetic and pharmacodynamics • Rapidly screen drug candidates	• Defect in drug detoxification	(168–170)
Murine	• Highly conservative pharmacokinetics • Conducive to curative effect screening • For interventional preclinical studies • Various strains for drug therapy	• Long life cycle • High animal costs	(171–174)

**Figure 7:**
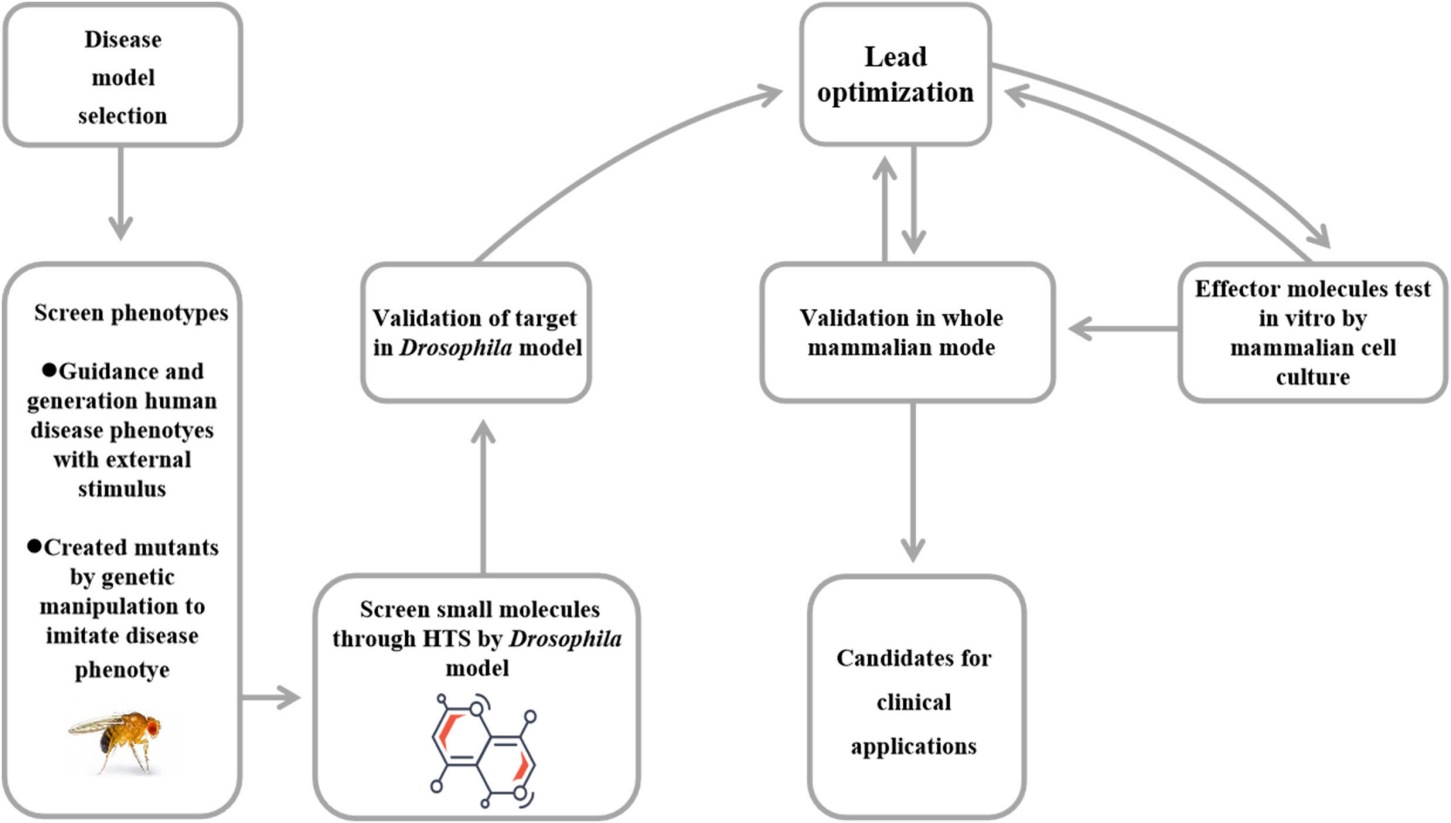
The flow diagram of *Drosophila* in drug screening. First, *Drosophila* model is generated in different ways. Screening of small molecules uses HTS and then validates target factors. Effective molecules are tested and cultured in mammalian cells to validate target. Finally, identified effectors are confirmed in whole mammalian models. HTS, high-throughput screening.

The usage of the *Drosophila* model for screening potential drug targets is illustrated in **Figure 7**. Generally, comprehensive human disease phenotypes can be achieved in flies through two primary methods (13): i) external stimulation mediated by chemical or mechanical factors and ii) genetic manipulation achieved through the knock-in or knock-out of disease-related genes. Subsequently, disease-related fly models are exposed to drug candidates, and relevant phenotypes are assessed. After the initial screening, potential drugs are further validated using genetic approaches, genomic analyses, and bioinformatics methods in fly models. Ultimately, effective hits identified through a common drug discovery strategy known as Phenotypic Drug Discovery (PDD) are evaluated in whole-mammal disease models to assess their efficacy and safety (57, 154). Currently, reported findings from fly models have informed mammalian studies. These applications are based on conserved biological principles and provide evidence supporting consistent drug function across multiple models. For example, classic studies have shown that histone deacetylase (HDAC) inhibitors exhibit neuroprotective effects in the polyglutamine fly model, becoming a key focus for subsequent validation in mouse models and clinical exploration (155, 156). Another example is Nrf2, a drug target whose pathway was first identified in *Drosophila*, then validated in mice, and now shows potential for human therapeutic use (157, 158).

### 4.3 Filtrating pharmacogenomic markers for COPD using *Drosophila*

Pharmacogenomics has been applied to the field of COPD therapeutics to analyze individual genes that influence drug response, such as the accurate diagnosis of AATD and the targeted prescription of inhaled corticosteroids (ICSs) (159, 160). Among these, genetic variation correlates with pharmacogenomic phenotypes and may have a more significant influence on prognosis than on disease risk, particularly well-known single-nucleotide polymorphisms (SNPs) (161). A major challenge, however, is the lack of sufficient samples for COPD pharmacogenomic studies; for example, SNPs associated with bronchodilator response (BDR) have been difficult to identify due to this limitation (162). *Drosophila* models could serve as a biological filter for elucidating the complex relationships between genetic variations and pharmacogenomics in COPD. First, flies can increase sample sizes for studying homologous genes. Additionally, their high reproductive capacity produces a large number of specimens for data analysis. Correspondingly, the genetic variations persist in the progeny genomes of COPD patients, necessitating extensive transgenerational studies in pharmacogenomics (163, 164). Second, flies are easy to use for establishing numerous transgenic lines to analyze specific SNPs identified as clinical biomarkers. This allows researchers to determine the response of SNPs to different drugs, thereby verifying the role of genetic variations in drug efficacy and toxicity. Finally, studying fly genes involved in drug metabolism can enhance our understanding of these genes in human drug metabolism. For instance, the fly gene *Cyp18A1*, a conserved member of the cytochrome P450 family expressed in the airway epithelial cells, can be used to study drug metabolism during cigarette smoke exposure (12, 165–167). These strategies allow SNPs identified in fly models to provide a prioritized shortlist of candidates for hypothesis generation and target ranking. The most promising COPD-related SNPs are then advanced to more costly and ethically sensitive mammalian models for final preclinical validation, thereby optimizing the path to clinical decision-making. These characteristics make flies a valuable model for predicting drug responses across diverse genetic backgrounds, positioning them as a preclinical and prioritization platform for investigating the robustness of drug targets against genetic variability.

## 5 Conclusion

*Drosophila melanogaster* has become a powerful system for interrogating COPD-relevant biological events *in vivo*, offering genetic tractability, easily visualized tracheal morphology, lower costs, and fewer ethical constraints. The molecular properties and cellular functions of the fly’s airway-enriched homologs are highly conserved with those implicated in COPD, enabling precise genetic perturbations to uncover mechanisms and model COPD phenotypes rapidly and at scale. Although *Drosophila* is not a direct substitute for clinical decision models, it serves as a valuable preclinical and prioritization tool to bridge the gap between genetic association studies and functional validation in vertebrate systems. Additionally, flies have emerged as a practical high-throughput platform for target discovery and drug screening, facilitating pharmacodynamic hypothesis testing before vertebrate studies.

Despite the conservation of biological processes between flies and mammals, several limitations restrict the extent to which the *Drosophila* trachea model can replicate the complex context of COPD: i) differences in smoke exposure—mammals are nasal breathers, whereas flies experience passive smoke exposure rather than active smoking. Humans utilize mouth-only devices, while flies exposed to cigarette smoke are more representative of second-hand smoking (175). ii) Differences in airway complexity—flies lack alveoli, submucosal glands, and a mucus-rich mucociliary apparatus. Gas exchange occurs through tracheoles rather than alveolar units, which prevents the induction of chronic bronchitis symptoms such as emphysema, mucus hypersecretion, and mucociliary dysfunction (37). iii) Differences in pharmacological responses—the effects of the same drug in flies can differ substantially from those in mammals, exhibiting off-target effects and unexpected systemic reactions, such as differences in caffeine metabolism between flies and mammals (176). Pharmacokinetic factors, including polymorphic isoenzymes of the P450 family, the complex regulatory network governing metabolism under stress, and heterogeneous drug distribution across tissues, primarily contribute to these pharmacodynamic differences between flies and humans (176–178). iv) Differences in the immune system—specifically, the absence of adaptive immune responses limits the interpretability of fly models for COPD studies, where adaptive immunity plays a dominant role in aspects such as chronicity, tissue destruction, and specific pathology.

Given that physiological limitations in *Drosophila* may restrict the development of COPD therapeutics, mitigation strategies must be implemented to bridge these gaps. For example, fly exposure rigs should be standardized using aerosol physicochemical properties and dose metrics to quantify cigarette smoke inhalation. In addition, employing co-infection models that mimic clinical trajectories can better approximate COPD exacerbations in patients. Concurrently, the biological characteristics of flies can be integrated with multi-omics, artificial intelligence technologies, and gene therapy to establish advanced predictive models that enhance interpretability. Furthermore, the side effects and optimal drug delivery methods for the COPD-related medications have not been thoroughly evaluated; subsequently, screening consequences outcomes must differentiate among various model types. Utilizing multiple models collectively to study COPD treatment will improve screening accuracy and advance personalized patient therapies in the future.
